# Risk ON / Risk OFF: Risk-Taking Varies with Subjectively Preferred and Disliked Music

**DOI:** 10.1371/journal.pone.0135436

**Published:** 2015-08-24

**Authors:** Marja-Liisa Halko, Markku Kaustia

**Affiliations:** 1 Department of Economics, School of Business, Aalto University, Helsinki, Finland; 2 Department of Finance, School of Business, Aalto University, Helsinki, Finland; University of Pennsylvania, UNITED STATES

## Abstract

In this paper we conduct a within-subjects experiment in which teenagers go over 256 gambles with real money gains and losses. For each risky gamble they choose whether to participate in it, or pass. Prior to this main experiment subjects identify specific songs belonging to their favorite musical genre, as well as songs representing a style they dislike. In the main experiment we vary the music playing in the background, so that each subject hears some of their favorite music, and some disliked music, alternating in blocks of 16 gambles. We find that favorite music increases risk-taking (‘risk on’), and disliked music suppresses risk-taking (‘risk off’), compared to a baseline of no music. Literature in psychology proposes several mechanisms by which mood affects risk-taking, but none of them fully explain the results in our setting. The results are, however, consistent with the economics notion of preference complementarity, extended to the domain of risk preference. The preference structure implied by our results is more complex than previously thought, yet realistic, and consistent with recent theoretical models. More generally, this mechanism offers a potential explanation to why risk-taking is known to change over time and across contexts.

## Introduction

Economics aspires to explain human behavior by the notion of individual utility maximization. While many specifications of utility functions have been used, some of which lead to what seems like strange or even unwise behavior, economists nevertheless tend to assume that an individual’s chosen actions follow their best self-interest. For example, in a classical paper Becker and Murphy explain behavior such as the use of heroin in a rational utility-maximizing framework [1]. Psychological approaches to decision-making, on the other hand, tend to assume that people make mistakes, and can thus choose what is not in their best self-interest. The topic of decision-making under risk, in particular, has seen an increasing number of studies in both camps. The interest is largely driven by the finding that risk preferences seem to be unstable across contexts [2–3]. This instability can be due to a behavioral bias, such as a framing effect [4–5], or driven by mood effects [6–7]. Alternatively, seemingly inconsistent behavior could at least partly be due to preference interactions [8–11]. Simple examples of preference interactions are pairs of complementary consumption goods, such as french fries and ketchup, or coffee and cream. Analogously in the context of risk-taking, a decision to take risk could give more (or less) utility depending on simultaneously experienced utility from other sources.

In this paper we introduce the idea of preference interactions as an explanation to changing risk-taking behavior, and utilize an experimental setup that helps disentangling the preference-based explanation from the alternative explanation of a psychological bias. Such risk preference interactions may be particularly relevant in domains that simultaneously involve risky decisions and entertainment value. Examples include gambling, extreme sports, and recreational driving by young adults. In finance, active yet unprofitable trading by some individual investors fits this description [12–14].

To test for preference interactions in risk-taking we utilize the revealed preference of each subject for selecting stimuli, which in the context of our experiment is music. By necessity, we employ a within-subject experimental setting, which also gives more power to identify the effect compared to a between-subjects design. As experimental subjects we recruited 25 teenagers, aged between 12 and 17 years. The number of subjects is in the typical range among decision-making studies that require subject-specific arrangements. The ample within-subject variation that is available provides enough power to identify the effects we are testing for. We chose teenagers for two reasons. First, we expected them to be good at explicitly identifying their specific musical taste. Several authors (see, e.g. [15–16]) consider teenagers the most fanatic music adepts of all age groups. For example, British adolescents to listen to music for an average of 2.5 hours per day [17]. For these reasons, teenagers may have more polarized, or at least more clearly defined musical tastes. However, this does not necessarily mean that the effects would be weaker with adults–music may even be more significant later in life [18–19]. Second, it is easier to create effective monetary incentives due to the subjects’ low level of income. The loss amounts from a single gamble ranged from 0.5 to 2 euros, and the win amounts ranged from 2 to 4 euros. These amounts are roughly comparable to a couple of days’ worth of disposable income for the average subject.

Prior to the main experiment, each subject identifies four pieces of what they consider their favorite music, as well as four musical pieces that they dislike. In this data several artists appeared on both ends of the preference scale, depending on the subject. For example, rap artists such as 50 Cent and Eminem were featured as the preferred music for some subjects, while at the same time representing most disliked music of some other subjects. The same is true also for pop / R&B artist Rihanna and the hard rock band Guns N’ Roses: they were among the favorites as well as the most disliked artists. Music by these artists appears to affect different people in different ways, consistent with [20–21]. Most important for our purposes, we know exactly which musical pieces each subject likes, and what they dislike. We utilize this revealed preference in the main experiment in connection to risky choice.

In the main experiment we measure risk-taking using binary-outcome, constant probability (50–50) gambles. We alternated the music being played in the background while the subjects were choosing whether to participate in the gambles. Each subject was played music from their personal favorites list (4 different songs), as well as music that they despised (4 different songs). In addition to the liked and disliked music, choices were also made under silence. We also varied the gain and loss payoffs of the gambles. This resulted in variation in the expected values of the gambles as the probabilities were held fixed. Each subject went through 256 gambles with different payoffs, and we identify the effects from within-subject variation in the gamble acceptance rates.

**Fig 1:**
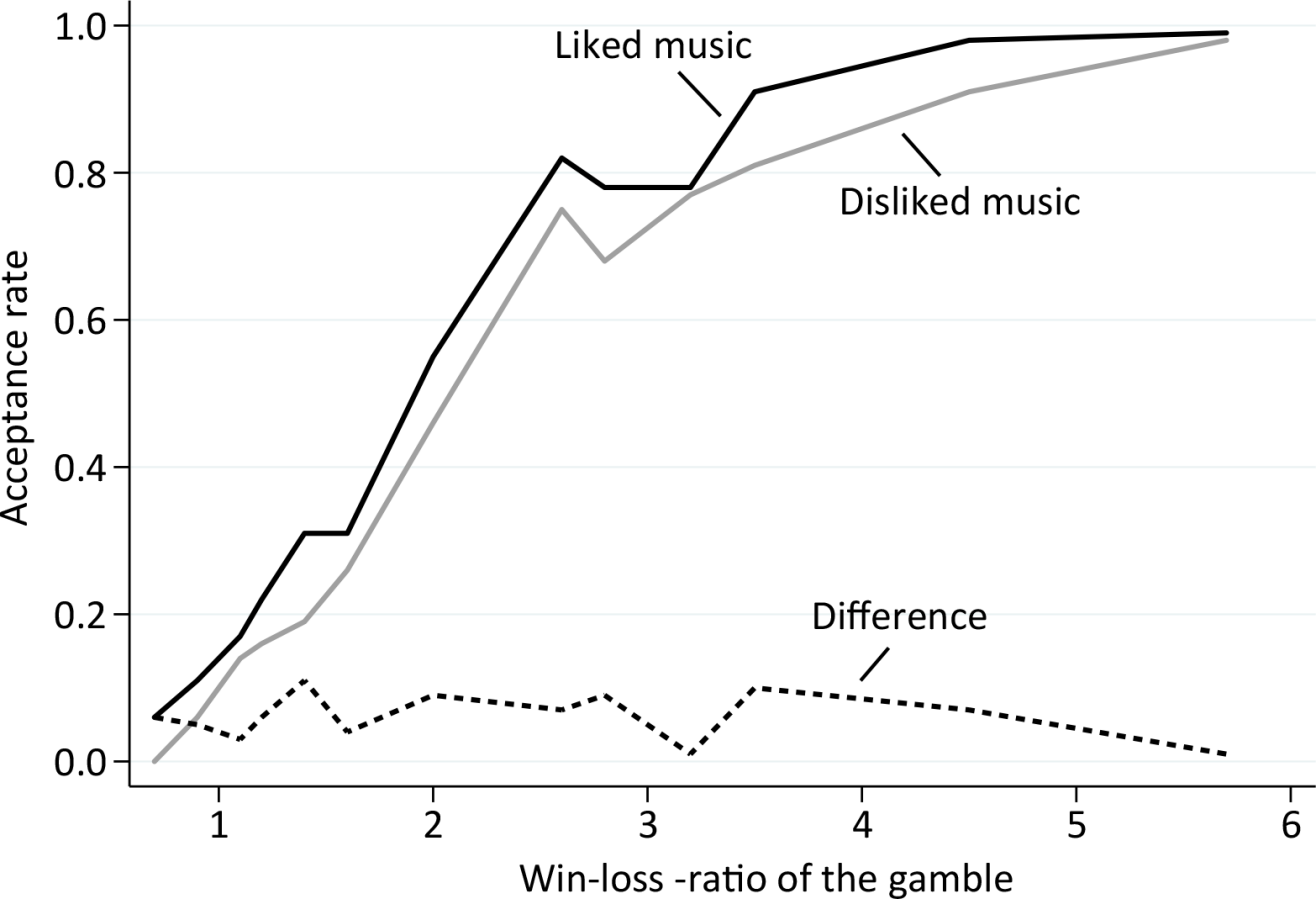
Gamble acceptance rates. Acceptance rates under liked music (black solid line) and disliked music (gray solid line), and their difference (dotted line) as a function of the win-loss–ratio of the gambles. The win-loss–ratio is formed by dividing the potential win amount by the potential loss amount.

We find that favorite music increases risk-taking, and disliked music depresses risk-taking relative to the baseline of no music. The musical style per se does not matter for risk-taking. Rather, the key factor is whether or not the subject’s personal musical taste and the music playing in the background are congruent. The frequency for accepting a gamble is 54.1% for favorite music versus 47.4% for disliked music. When no music was playing the acceptance rate is 51.4%. The effects of music are evident in all kinds of gambles but the difference in acceptance rates in favor of liked versus disliked music is greatest (about 10 percentage points) when unconditional acceptance rates are around 50%, which happens with a ratio of gains to losses around 2:1 (see Fig 1). The positive effect of favorite music and the negative effect of disliked music are both statistically significant at the 5% level under various alternative estimation methods. In particular, they are present in a logit regression with subject fixed effects, in a random effects regression including surveyed subjective risk attitude as a control variable, and in the distribution of coefficients from subject specific regressions, when controlling for the gain and loss amounts of the gambles.

These results are consistent with preference interactions of the type described by [8–10] and imply that such effects can extend to risk preference, in addition to preferences for consumption goods. It appears as if listening to preferred type of music increases the marginal utility of money. Such preference structures can create behavior that is observationally equivalent to unstable preferences when viewed through the standard economic model in which the marginal propensity to take risk is independent of utility derived from other sources. However, time varying risk-taking behavior can be consistent with utility maximizing behavior in a more complex preference structure. In subsequent work involving neuroimaging methods, we find that the behavioral effect of music on risk-taking co-varied with brain activation in amygdala and dorsal striatum [22]. This evidence is consistent with our preference-based interpretation as these brain areas are known to be key components of the value computation and coding of preference information [23–24]. The preference-based interpretation is also consistent with the neuroimaging results of [25] showing that the activation in the reward areas of the brain are proportional to subjective ratings of music.

A justified question is whether the results could arise through decision-psychologic influences, as opposed to preference interactions. Prior research has proposed at least four mechanisms that mediate the effect of mood on risk-taking: mood maintenance hypothesis, subjective probability weighting, affect infusion, and impact on cognitive processing strategies. We defer a more detailed discussion of the psychological mechanisms to the discussion section of the paper, where we argue that none of these theories can adequately account for our findings.

## Methods

We recruited 25 adolescents (aged 12–17 years, 12 males, 13 females) in Helsinki, Finland, by announcing an invitation to participate in an experiment that concerns music and attitude towards monetary gains and losses. Participants were told that the experiment consists of two separate sessions (S1 Experimental Instructions). For the first session they were asked to select, and bring with them four pieces of their favorite music and four pieces of music they disliked (on music CDs or mp3-files). We recognize the possibility that locating and bringing in the disliked music requires more effort than to do the same for favorite music. If this is the case the impact of disliked music could be understated in our empirical tests.

The announcement also explained the payment structure: the subjects would be paid 10 euros for participating in the first session, while earnings in the second session would depend on the decisions they make during the experiment as well as chance outcomes. The subjects could either win or lose money in the second session. We adopted this two-session structure based on the recommendation by [26]. The idea is to make the subjects feel that they would face actual potential losses in the gambles, and less likely to feel as if they were “gambling with the house money”. The maximum amount that the subjects could win was 20 euros, and the maximum amount they could lose was 10 euros. A written informed consent was solicited from the subjects’ parents prior to the experiment.

### Session 1

The eight pieces of music that each subject brought with them were first copied onto a computer for later use in the second session. The subjects then filled out a questionnaire surveying their risk attitude and some background information. The date of the second session was agreed on, leaving at least one week between the sessions. The fixed payment of 10 euros was paid and the subjects were reminded that in the second session they would participate in a computerized experiment in which one can either win or lose money.

### Session 2

**Fig 2:**
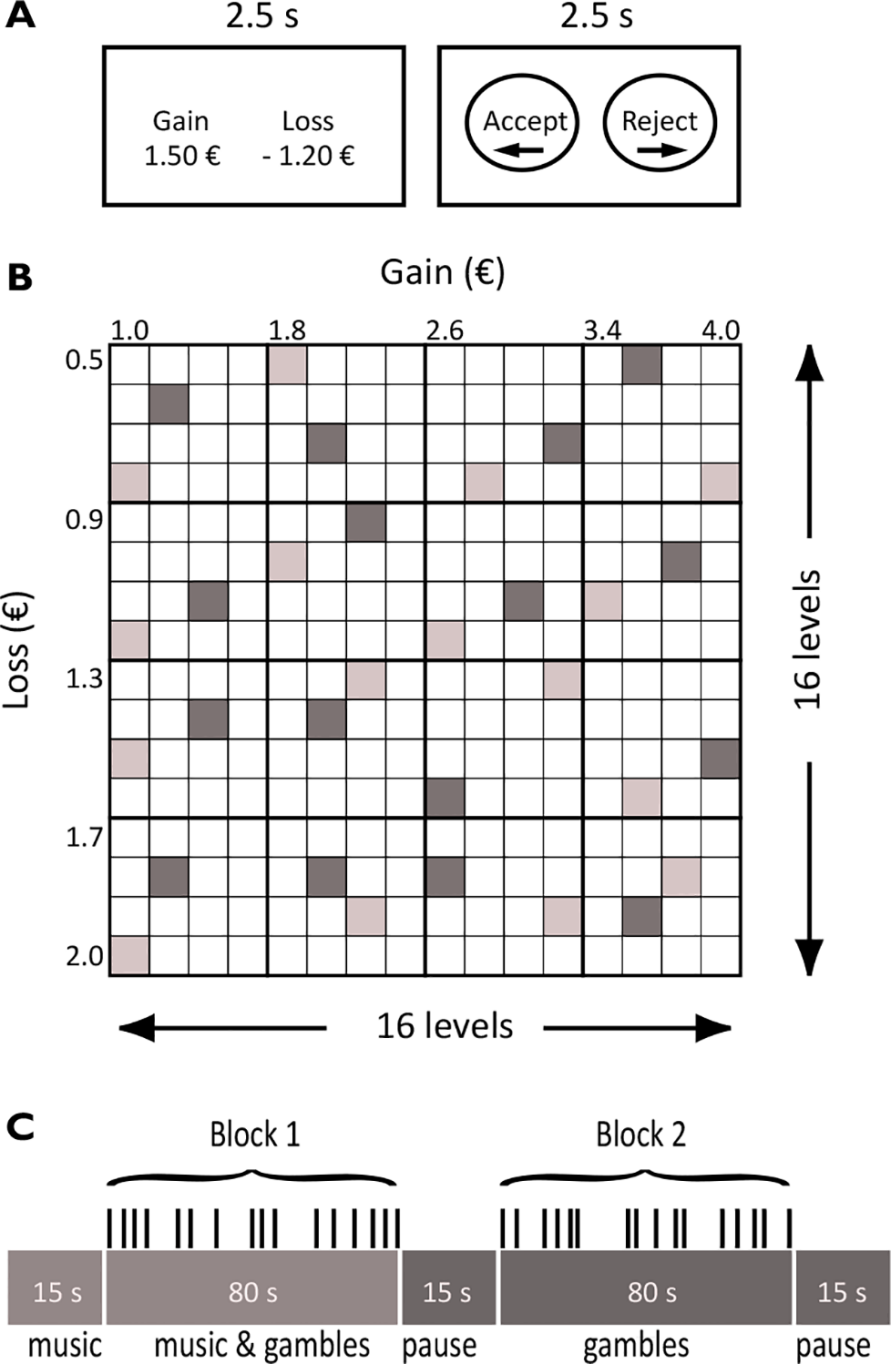
The financial decision making task. (A) Exemplary computer screen plot from the experiment. The task of the subjects was to accept or reject gambles that offered a 50–50 chance of gaining or losing money. Gains ranged from 1 to 4 euros and losses from 0.5 to 2 euros. (B) The payoff matrix comprised 256 different gambles. The 256 gambles were divided into 16 sets of 16 gambles each. Within a set, the 16 gambles were scattered around the payoff matrix such that only one gamble came from each of the separate 4 by 4 areas in the matrix. The two different shades of grey rectangles in the figure represent two examples of a set of gambles. (C) Subjects played 16 different blocks, 16 gambles in each block, and a block with music was always followed by a block without music. To keep subjects’ attention high and to avoid unreflected automatic response we varied the length of the interval between the gambles (from 0.5 to 3.5 seconds).

In the main experiment, which took place after at least a week had passed since session 1, the task of subjects was to accept or reject gambles that offered a 50–50 chance to win or lose money. Accepting a gamble, for example [1.50, –1.20], meant that the subject was willing to participate in a gamble that offered a 50% change of winning 1.50 euros and a 50% chance of losing 1.20 euros. The experiment was conducted individually in a private room using a computer program. Fig 2A shows the computer screen plot for the aforementioned example. The gain and loss amounts were shown for 2.5 seconds, after which the subjects had 2.5 seconds to choose “accept” or “reject” by pressing designated buttons on the computer keyboard. After deciding on the gamble, there was a break of 0.5 to 3.5 seconds until the next gamble was shown. We varied the length of this break to avoid unreflected automatic responses and to keep the subjects focused.

The results of the gambles were not shown during the course of the experiment. Knowledge on prior gains or losses can influence behavior [4, 27]. While these effects are interesting in their own right, for the purposes of this paper they represent a confounding effect that we wanted to avoid. At the start of the experiment the subjects were informed that five randomly determined gambles would be played for real at the end of the session. There were 16 different win outcomes ranging from 1 to 4 euros, and 16 loss outcomes ranging from 0.5 to 2 euros. Each subject went through the full payoff matrix and none of the gambles were repeated. This corresponds to 16 x 16 = 256 gambles (Fig 2B).

Gambles were presented under three conditions: while the subjects’ favorite music was playing (64 gambles), while disliked music was playing (64 gambles), and gambles with no music playing (128 gambles). Sixteen gambles under one condition were assembled into a block of 80 second duration. Each block of music was followed by a block without music. The order of blocks was either L-L-D-D-L-L-D-D- or D-D-L-L-D-D-L-L- (where L stands for liked music, D for disliked music, and—for no music), counterbalanced across subjects (Fig 2C). During all conditions gambles were randomly drawn from the payoff matrix. We applied random sampling that ensured an even mix of different types of gambles under all three conditions (Fig 2B).

The complete session lasted about 40 minutes. At the end, five gambles were randomly drawn, and the ones that the subject had accepted were played for real. Payoffs were determined by the roll of a dice (values of one to three indicated a loss, and four to six indicated a win). The average total payment was 13.80 euros (SD = 3.93) which is, according to our survey, somewhat more than the average weekly disposable income for our subjects.

The data of two subjects were discarded: one due to misunderstanding the task, and one due to a technical error with the music that was played. The analysis is thus based on data for 23 subjects, 12 females and 11 males, mean age 14.6 years, SD = 1.62, range 12–17 years.

## Main Results

**Fig 3:**
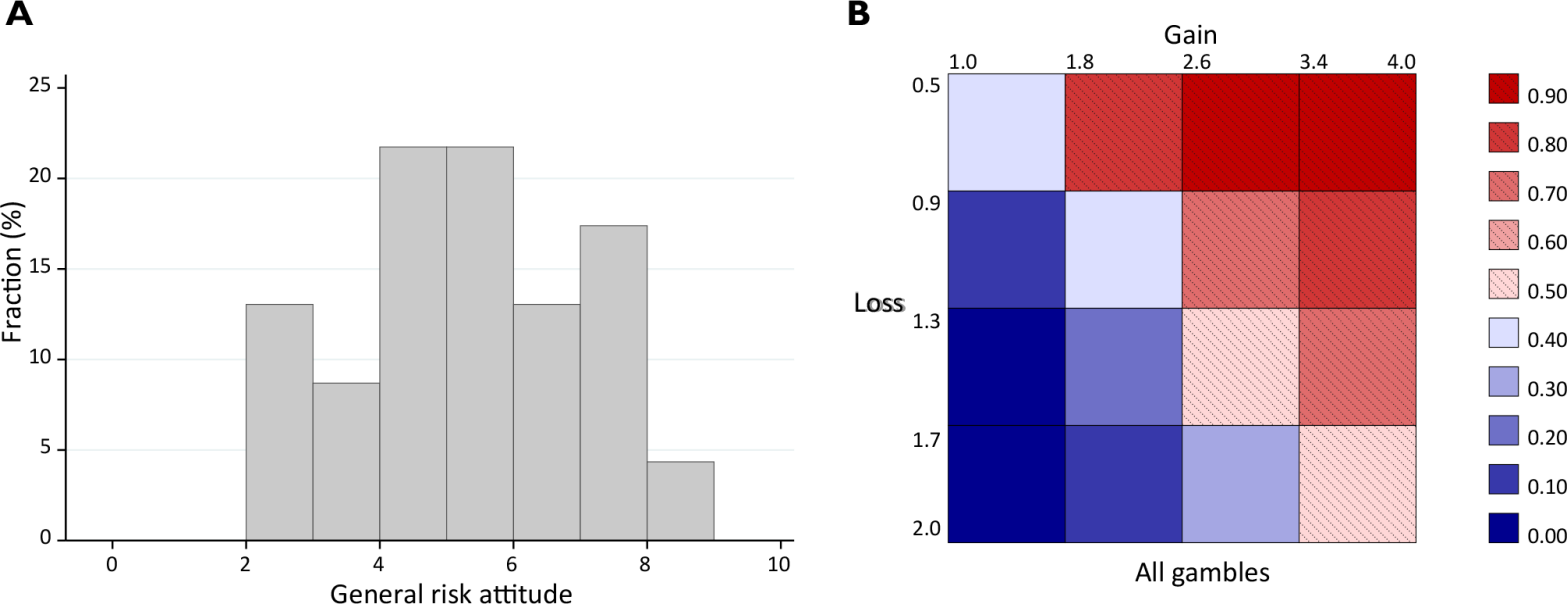
Risk attitude and mean acceptance rates. (A) The distribution of risk attitude; 0 = not willing to take risk, 10 = completely willing to take risk. The average willingness to take risk was 5.07 (SD = 1.92). (B) Payoff matrix and mean acceptance rates, all gambles.

In the first session of the experiment, the subjects filled a questionnaire where we surveyed their risk attitude. They first rated their general willingness to take risk on a visual analog scale, that is, by indicating a position along a horizontal line, 10 cm in length, with the end-points labeled ‘Not willing to take risk’ and ‘Completely willing to take risk’. The distribution of the responses to the general risk question is depicted in Fig 3A. The average willingness to take risk was 5.07 (SD = 1.92).

A risk neutral subject would be indifferent between participating in and rejecting a gamble with a win-loss ratio of 1:1. Even a normal risk-averse decision maker, provided that he or she is optimizing lifetime consumption and is not pathologically risk-averse, would accept a small-scale gamble as long as the win-loss–ratio is slightly over 1 [28]. However, experimental studies show that people generally tend to be more sensitive to losses than to gains (loss aversion). Indifference between accepting and declining equiprobable binomial gambles typically obtains when the ratio of the win amount to the loss amount is 2:1 [29–30]. Fig 3B illustrates the distribution of the acceptance rates for the gambles for different combinations of gains and losses in our experiment. The acceptance rates are around 50% along the diagonal where the ratio of the win amount to the amount of loss is 2:1, and decrease when moving towards less favorable gambles. These results show that our adolescent subjects do not differ from the general population in their degree of loss aversion, even if adolescents are known to engage in some types of risky activities more often than adults [31].

**Fig 4:**
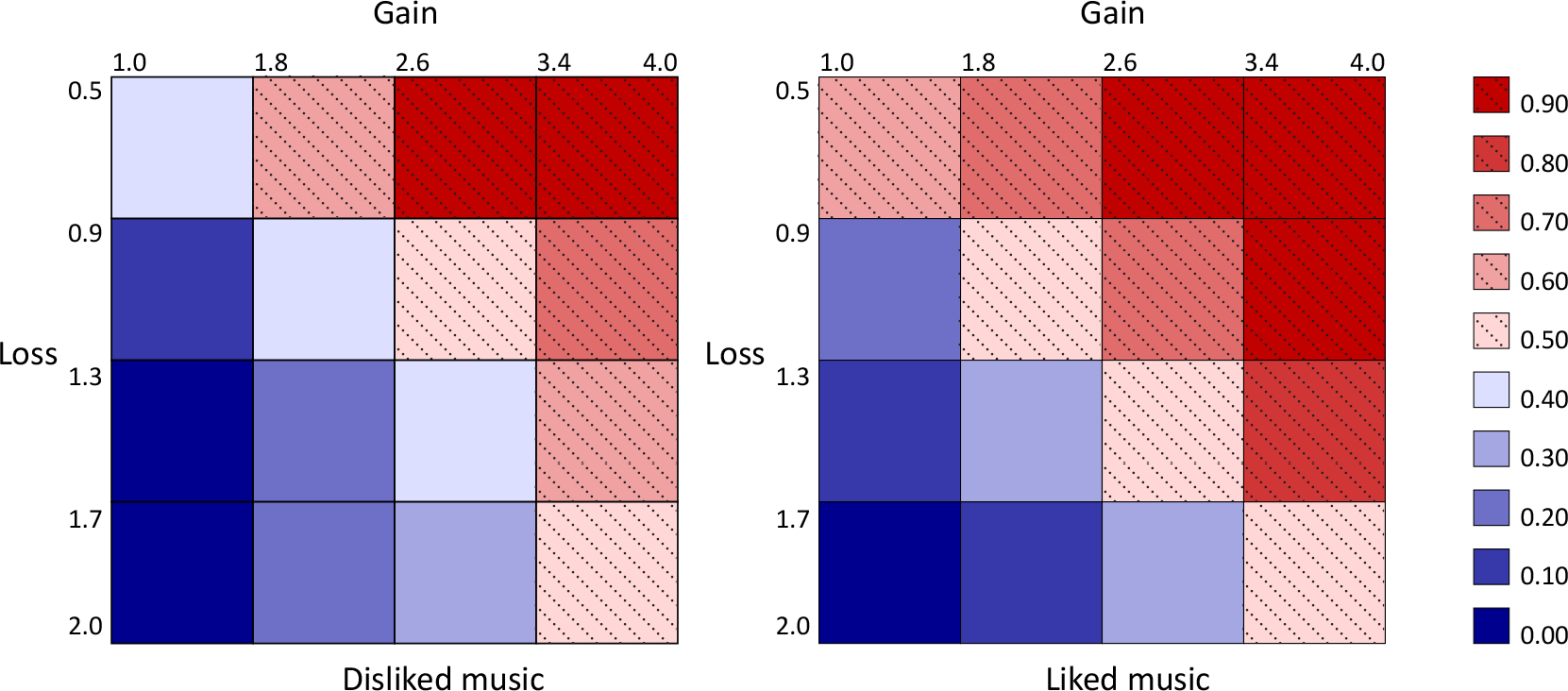
Interaction between music and risk taking. Payoff matrix and mean acceptance rates in two conditions: subjectively disliked music and liked music. In a 2 by 4 by 4 ANOVA (music, gain, loss), the main effect of music (disliked vs. liked) on the mean acceptance rate was statistically significant (F(1,22) = 12.04, p = 0.002), likewise main effects of gain (F(3,66) = 102.04, p < 0.001) and loss (F(3,66) = 94.47, p < 0.001). As expected, the interaction was between gain and loss was significant (F(9,198) = 5.09, p < 0.001); none of the interactions with music was significant.

Table 1 shows the impact of music on the tendency to participate in gambles. Univariate results reported in Panel A show that the frequency for accepting a gamble is 47.4% for disliked music versus 54.1% for favorite music. When no music was playing the acceptance rate is 51.4%. Favorite music thus alleviates, and disliked music exacerbates loss aversion. Fig 1 graphs the acceptance rates as a function of the win-loss–ratios of the gambles. The effect of music is evident in all kinds of gambles. The difference in acceptance rates in favor of liked versus disliked music is about 10 percentage points along the diagonal of the payoff matrix, that is, when the ratio of gains to losses is 2:1 (Fig 4). The mean effects of favorite and disliked music, compared to no music, are statistically significant at the 5% level under various estimation methods which we describe in detail in the multivariate analysis section.

**Table 1:** The effect of music on risk-taking, statistical tests.

Panel A.	Favorite music	No music	Disliked music
Mean acceptance rates	0.541	0.514	0.474
Difference compared to ‘No music’	0.027		-0.040
Difference between ‘Favorite music’ and ‘Disliked music’		0.067	
Panel B. Regressions with all subjects	Favorite music		Disliked music
Linear probability model (OLS)	0.034**		-0.034**
	2.16		-2.16
OLS with subject fixed effects	0.034**		-0.033**
	2.32		-2.31
Logit, conditional subject fixed effects	0.167***		-0.165***
	3.04		-3.32
Logit, subject random effects	0.167***		-0.165***
	2.84		-3.36
Panel C. Subject-specific logit regressions	Favorite music		Disliked music
Mean coefficient	0.169**		-0.168**
	2.51		-2.62

**Panel A** shows the mean acceptance rates of binary gambles in which subjects (N = 23) could either win or lose money with equal probability. The number of observations for each subject is 256, of which 128 are for ‘No music’, 64 are for ‘Favorite music’, and 64 are for ‘Disliked music’. **Panel B** shows results from four different regression models testing the effect of the musical condition on the decision to accept the gamble. T-statistics (z-statistics for logit regressions) are presented below the coefficients. In calculating the t-statistics we use standard errors robust to heteroskedasticity in all analyses. For fixed effects and random effects models such standard errors are obtained with bootstrapping. **Panel C** shows results from running separate Logit regressions for each subject (256 observations in each regression), and taking averages of the subject-specific coefficients. The t-statistics in Panel C are from a standard t-test of means. All regressions include the expected value of the gamble as a control variable (not reported). Statistical significance at the 5% and 1% level are indicated by ** and ***.

One way of judging the magnitude of these effects is to consider an offsetting change in a gamble’s loss amount that is needed for keeping the acceptance rate statistically equal, while music is being varied. Calculated in this way, liked music offsets a 3.1% increase in the loss amount, and disliked music offsets a 4.6% reduction in the loss amount. These magnitudes are economically relevant. The effect varies for different types of gambles. Liked music only slightly further increases acceptance rates among the most favorable gambles (above the payoff matrix diagonal) where acceptance rates are already high, but disliked music still further lowers the acceptance rates in the least favorable gambles (below the payoff matrix diagonal). In these least favorable gambles the acceptance rate is only 16%. However, the impact of music, again judged against an offsetting loss amount, is very large: a change of 25–30% in the loss amount is required to statistically offset the impact of either liked or disliked music on the acceptance rate. These results suggest that musical taste may interact with taste for risk particularly strongly in high-risk situations.

The effect magnitudes are similar for both genders, and the difference between favorite and disliked music statistically significant (p-value 0.01). Boys were slightly more influenced by disliked music, and girls slightly more influenced by favorite music, but these differences are not significant. The unconditional acceptance rate declines over the course of the experiment in an approximately linear fashion through blocks 1 to 16. It is 52.0% on average for the first half (blocks 1–8), and falls to 45.8% for the second half (blocks 9–16). This may reflect decision fatigue, and the associated tendency to ‘do nothing’, which in our experimental setup favors declining the gamble. It can also be due to a kind of mental risk budgeting: as the experiment progresses the subjects may start feeling that they have already taken enough risk, though in reality the risks do not accumulate in this way: only a fixed number is being played out for real at the end. Also the effect of music is slightly reduced for the latter half, but it nevertheless remains marginally significant (p-value 0.07) even when the sample size is cut in half.

## Multivariate Analysis

To assess the statistical significance and robustness of the result we estimate three types of regression models and implement two alternative assumptions about unobserved heterogeneity. We estimate all regressions with maximum likelihood and use standard errors that are robust to heteroskedasticity. Using all methods we find that subjectively liked music increases risk-taking, while disliked music decreases risk-taking, and the results are statistically significant at the 1–5% level.

We start by estimating the propensity to accept a gamble using a linear probability model (OLS regression). The dependent variable *y*_*ij*_ is a binary choice variable representing an acceptance (“1”) or a rejection (“0”) of gamble *j* ∈ {1,…,256} by subject *i* ∈ {1,…,23} and the model is

$$y_{ij}=\alpha +\beta_{E}E_{j}+\beta_{G}G_{ij}+\beta_{B}B_{ij}+\varepsilon_{ij}$$ (1)

where *E*_*j*_ is the expected value of gamble *j*, *G*_*ij*_ and *B*_*ij*_ are zero-one indicator variables for subjectively liked (“Good”) and disliked (“Bad”) music, respectively, being played while subject *i* was considering gamble *j*. The betas, *β*, represent the coefficients to be estimated with subscripts corresponding to the variables, and *α* is the constant term. As an alternative specification to using the expected value, *E*, to model the attractiveness of the gamble we include the amount to win and the amount to lose as two separate variables in all the models. The results are similar. Panel B of Table 1 reports the results from estimating (1), yielding coefficients of 0.034 for *G* (*t*-value 2.16) and –0.034 for *B* (*t*-value –2.16).

We then add subject specific regression constants, i.e., estimate a fixed effects linear probability model

$$y_{ij}=\alpha +\beta_{E}E_{j}+\beta_{G}G_{ij}+\beta_{B}B_{ij}+c_{j}+\varepsilon_{ij}$$ (2)

where *c*_*i*_ is the subject specific regression constant which captures between-subject variation in the tendency to participate in the gambles. Estimating (2), as reported in Panel B of Table 1, yields coefficients of 0.034 for *G* (*t*-value 2.32) and –0.033 for *B* (*t*-value –2.31).

An analysis of a dichotomous dependent variable is frequently conducted with a logit model, an approach that we also follow. However, the interpretation of coefficients is less straightforward, and fixed effects estimation may cause problems, compared to the linear probability model. Our logit model relates the probability of subject *i* accepting gamble *j* to the explanatory variables as follows

$$ln\left(\frac{p_{ij}}{1-p_{ij}}\right)=\alpha +\beta_{E}E_{j}+\beta_{G}G_{ij}+\beta_{B}B_{ij}+\varepsilon_{ij}$$ (3)

where *p*_*ij*_ is the probability that *y*_*ij*_ = 1 conditional on the explanatory variables. We estimate two types of unobserved effects models.

First, a conditional fixed effects logit which additionally conditions the probabilities on a subject specific count of accepted gambles $Y_{i}=\sum_{j=1}^{256}y_{ij}$. We obtain robust standard errors by bootstrapping (50 repetitions). Panel B of Table 1 shows coefficients of 0.167 for *G* (*z*-value 3.04) and –0.165 for *B* (*z*-value –3.32).

Second, we estimate a subject random effects model. Maximum likelihood gives consistent and efficient estimates of a random effects model assuming that the unobserved effects do not correlate with the explanatory variables. As a control variable we include the subjects’ general risk attitude that was surveyed in the first part of the experiment, about a week prior to the main experiment. In this model we effectively assume that the probability of subject *i* accepting gamble *j* is as follows

$$p_{ij}(y_{j}=1)=f(E_{j},G_{ij},B_{ij},R_{i},\alpha_{i})$$ (4)

i.e., related to gamble characteristics, *E*_*j*_, the musical condition prevailing during the decision, *G*_*ij*_ and *B*_*ij*_, the subject’ general risk attitude, *R*_*i*_, as well as the subject specific random effect, *α*_*i*_, and that the random effect remaining after controlling for *R*_*i*_ is independent of the explanatory variables. One can think of these random effects as deviations from the subjects’ baseline risk attitude, arising from day-to-day variation, or from differences in context specific reactions. We again use bootstrapped standard errors. Panel B of Table 1 shows coefficients of 0.167 for *G* (*z*-value 2.84) and –0.165 for *B* (*z*-value –3.36).

We also run a standard logit model for each subject separately estimating coefficients for the musical condition dummies and controlling for the expected value of the gamble. Each regression thus has 256 observations corresponding to the number of all different gambles. The coefficients for liked music are positive for 83% of the subjects and the coefficients for disliked music are negative for 74% of the subjects. The means of the coefficients across all subjects are similar to the coefficient estimates obtained by the other methods (0.17 for *G* and –0.17 for *B*), and the median values of the coefficients are somewhat larger in absolute magnitude. We test for statistical significance of the averages of the individual *G* and *B* regression coefficients with a standard *t*-test and obtain *t*-values of 2.51 (for *G*) and –2.61 (for *B*). These results are reported in Panel C of Table 1.

## Discussion

This section evaluates potential underlying psychological mechanisms, as well as discusses the theoretical implications of the results.

### Psychological mechanisms

Our thesis in this paper is that a taste of risk in one moment in time is affected by simultaneous utility from other sources. This is a classical economics-based argument—tastes matter—but departing from the classical specification of simple one-dimensional utility of wealth. In the experiment we used music as this other source of utility. A natural question to ask is whether psychological theories, whereby mood affects risk-taking, could also explain our findings. Below we consider the most prominent of such theories. While they may have some merit here, it appears that none can fully account for the effect in our setting. To facilitate the link to the literature on mood effects, we assume below that listening to favorite music elevates mood, at least compared to listening to disliked music [22].

Mood maintenance theory—also referred to as mood regulation—says that people in good mood have more to lose compared to people in bad mood, and thus avoid taking risks with potential negative consequences that could erode their good mood [32–36]. Contrary to this idea, we find that people take more risk while listening to their favorite music.

Subjective probability weighting can also be responsible for mood effects in risk-taking. People on positive moods generally assess bad outcomes as being less likely compared to people on negative moods [37–38]. Such probability weighting is known to matter at the ends of the probability scale, and is not an issue with the constant 50% probabilities we use [39–40]. It is thus unlikely that mood changes would impact the perception of probabilities in our experiment.

The affect infusion model describes the process of selecting, learning, and interpreting new information about a risky situation, and incorporating it into existing knowledge and experiences [41]. Under this theory, good mood should increase risk-taking if it primes access to memories of mood congruent outcomes from earlier risky choices. Our simple binomial constant probability gambles do not readily fall under the domain of affect infusion theory as they do not involve the complex information processing that it assumes.

Mood states can also interplay with the type of cognitive processing strategies utilized, which might mediate the impact of mood on risk-taking. Specifically, people may be more likely to employ analytical problem-solving under negative moods, while more likely resorting to heuristics that lead to behavioral biases under positive moods [42–43]. In contrast to this idea, we find that subjects listening to their favorite music perform closer to the normative benchmark of participating in all the gambles. That is, good music makes the subjects less loss-averse, and less biased in that sense.

Finally, we note that classical Pavlovian conditioning, or a ‘hedonic forecasting mechanism’ leading to a biochemical response to cues and rewards [44–45] is not possible in the context of our experiment. This is because in our experiment the gambles are only played at the end, so the outcomes can not affect choices. We nevertheless consider it quite plausible that conditioning can contribute to preference formation prior to the experiment. For example, consider a youngster who ventures to ask a girl of his dreams for a dance while a particular song is playing. An affirmative response could lead not only to liking the song, but also to associating the song with reward from taking risk. In any case, the subjects enter our experiment endowed with whatever preferences they have, and then behave consistent with preference interactions, even if Pavlovian conditioning has affected the formation of those preferences at some point earlier.

The overall conclusion is that prominent psychological theories on mood and risk-taking do not provide a compelling explanation to our results, at least when considered individually. However, neither does standard economic theory. The next section concludes by sketching a potential approach for modeling risk-preference interactions that allows a more general utility specification, as well as integrating more psychology into it. Incorporating affect infusion strategies and conditioning in a modified form may be fruitful. However, even if the phenomenon we document is potentially amenable to economic utility-based modeling, a whole other question is whether the subjects’ behavior can be explained in terms of utility maximization. Most axioms of expected utility theory have been violated in experiments ([46–47, 29, 48–51] and others). The bottom line regarding state-varying risk preferences is that to give expected utility theory a fighting chance, utility must be correctly specified. It is toward this goal that our paper takes an important step.

### Theoretical implications

How value is encoded in the brain remains a key question in neuroscience, microeconomics, and the study of learning [52]. Neuroeconomics supports the view that value and thereby preferences are formed as interactions among specialized components in the brain [53–57]. The relative strength of the activity in different brain areas varies by the type of the evaluation, such as absolute vs. relative to a reference point, time vs. risk, or undiscounted vs. discounted payoffs. It seems plausible that the evaluation of fundamentally different aspects, such as risk and music, might also modulate brain processes. Given the neuroeconomics view of preference formation, the modulation might then underlie the preference interaction. The neuroimaging results of [22] are broadly consistent with this conjecture, although they do not specifically test it.

One possible mediating channel for preference interactions is stress. Music can reduce stress. For example, the effect has been documented for surgery patients [58]. But music also has the capability of enhancing stress: experimental subjects playing a violent videogame (Quake III) with its built-in music turned on, showed a higher blood concentration of stress hormone cortisol compared to other subjects playing the same game in silence [59]. In risky decision-making, music and stress have been associated with similar changes in the way that rewards and punishments are evaluated in the brain [22, 60].

Recent experiments in economics could be interpreted in a preference-interaction framework [61–62]. Subjects viewing a scene from a horror movie choose a significantly lower certainty equivalent in a hypothetical lottery question [61]. However, this general pattern is broken when the sample is split by taste for horror movies. Based on the figures reported in the paper, we calculate that viewing the movie clip decreases the certainty equivalent for those who dislike horror movies by 35%, but increases the certainty equivalent for those who like horror movies by 81%, compared to subjects who are indifferent regarding horror movies. The bubble pattern typically obtained in experimental asset markets is much stronger if subjects had just viewed an exciting movie clip (vs. a dull documentary or a sad drama clip) before the trading session [62].

Some earlier evidence on the effect of music on consumption can also be interpreted as being supportive of preference interactions. Enjoyable music increases supermarket and restaurant sales [63–65]. Consumers also report higher overall satisfaction with their shopping experience when music and ambient scent in the store are congruent [66–67]. It has been found that most shoppers selected a French wine when French music was playing, but selected a German wine when German music was playing [68]. All these studies are consistent with musical preference interacting with the utility evaluation in another dimension. Regarding the interaction of music risky and behavior specifically, people increase their driving speed and engage in more red light violations when high tempo music is playing on the background [69]. However, driving performance while listening to music can be better for some driving tasks, such as car following [70].

We conclude this section with a discussion on modeling risk-preference interactions. As a starting point, consider the standard setup of state dependent utility [71–72]. The decision-maker’s preferences over uncertain options are said to be state-dependent if the prevailing state of nature affects his or her evaluation of the consequences. In our case, music constituted a state of the world which may have influenced the decision-maker’s preferences, for example, by altering his or her risk attitude. Let us assume that a standard Kahneman and Tversky–type utility function with a reference point zero represents the preferences or our subjects [29]. Assume further, for simplicity, that the utility function is piecewise linear. Music may then influence the preferences by changing the slopes of the segments of the utility function. Hence, hearing favorite music would increase the slope for positive outcomes and decrease the slope for negative outcomes.

As a slightly more complicated framework, consider the Loewenstein, O’Donoghue, and Rabin–setup of state dependent preferences [11]. The focus in [11] is on prediction errors of future utility arising from the decision-maker’s incomplete understanding of the effect that the state has on his or her utility. One could define utility over a gamble, represented by a probability distribution of outcomes, *x*, and music, *m*, as *U*(*x*,*m*), such that ∂U∂m≥0. Note that treating *m* as music is without loss of generality: *m* can be anything that can be enjoyed while making risky choices, say, visual stimuli. A special case of no forecast errors can be thought of as the fully rational benchmark. That is, subjects choose whether to take risk by maximizing *U*(*x*,*m*), understanding that whether *U*(*x*) > *U*(*x*′) depends not only on *x* and *x*′, but also on the value of *m*. Now given current musical state *m*, denote the decision-maker’s prediction of utility in a musical state *m*′ ≠ *m* by $\hat{U}(x,m\prime |m)$. With full rationality, $\hat{U}(x,m\prime |m)=U(x,m\prime )$. Allowing prediction errors *e*, that is, $\hat{U}(x,m\prime |m)=U(x,m\prime )+e$, is a possible avenue for integrating more psychology into this model of risk-preference interactions. For example, the decision-maker might extrapolate from the utility given current musical state, such that $\hat{U}(x,m\prime |m)>U(x,m\prime )$ when *U*(*x*,*m*) > *U*(*x*,*m*′). *U* could also be made habit dependent such that experiencing relatively more of a high (low) musical state leads to increasing (decreasing) risk-taking in all states. These features could be used for modeling a dynamically inconsistent agent who is subject to more surprises and more volatile preferences compared to the fully rational benchmark.

## Conclusion

Using an experimental setting which involves real money stakes, constant probabilities of winning, and within-subject alteration of the type of background music, we find that hearing one’s favorite music playing increases risk-taking, and disliked music suppresses risk-taking, compared to a baseline of no music. The difference in acceptance rates in favor of good music is about 10 percentage points along the diagonal of the payoff matrix.

We interpret these results as suggesting that preference complementarities extending beyond the realm of goods and services are possible. Listening to one’s preferred music increases experienced utility per se, and simultaneously increases the marginal utility of money. This increases the likelihood of participating in a gamble. Disliked music, on the other hand, would lead to a marginal utility of taking the gamble that is lower than what prevails under silence. The preference-based interpretation of the results is supported by recent research in neuroscience. The activation in the reward areas of the brain are proportional to subjective ratings of music [25], and the behavioral effect of music on risk-taking co-varies with brain activation in the amygdala and the dorsal striatum [22]–brain regions known to be key components of value computations.

Preference complementarities can explain time-varying risk preferences. Earlier studies have attributed such effects to behavioral bias. We wish to point out that these explanations are not mutually exclusive, however. How such preference complementarities would arise provides a topic for future research. Understanding the formation of preferences is a central issue in many disciplines.

## Supporting Information

S1 Experimental Instructions (PDF)

S1 Table A complete list of choices. (XLSX)
