# Supplementary material for: Risk ON / Risk OFF: Risk-Taking Varies with Subjectively Preferred and Disliked Music
Source: PLoS One. 2015 Aug 24;10(8):e0135436. doi: 10.1371/journal.pone.0135436 (PMC4547734; doi:10.1371/journal.pone.0135436)
Supplement: S1 Experimental Instructions — (PDF) [file pone.0135436.s001.pdf]

S1 Experimental Instructions.

General instructions for participants (Session 1)

You are participating in an experiment that is part of the research conducted at the School of Business. All the information you give will be handled confidentially and anonymously, meaning that the decisions you will make in this experiment will not be revealed to anyone and your name will not be connected with your choices. Please read the instructions carefully and do not hesitate to ask questions if there is something you don’t understand.

This is **the first session** of the experiment. At this session, we collect some background information, copy the music you have brought along, and agree the date of the second session of the experiment. It is very important that all participants take part in both sessions.

After this session, you will be paid in cash 10 euro. The second session of the experiment is related to decision-making involving monetary gains and losses. At the second session of the experiment, your earnings will depend on the decisions you make. At the maximum, you can win 20 euro, or at the maximum, you can lose 10 euro.

General instructions for participants (Session 2)

You are participating in an experiment that is part of the research conducted at the School of Business. All the information you give will be handled confidentially and anonymously, meaning that the decisions you will make in this experiment will not be revealed to anyone and your name will not be connected with your choices. Please read the instructions carefully, and do not hesitate to ask questions if there is something you don’t understand.

This is **the second session** of the experiment. This session is related to decision making and is conducted using a computer. During the experiment, you will hear music and make decisions.

The experiment includes several rounds. If a round begins with music, think whether you like the music or not. After a while, you are asked to evaluate the music you are listening, using the following scale:

Do you like the music?

Very much

Not at all

You can move the cursor using the arrow keys (← and →). After music evaluation, you are presented different gambles. In each gamble, you can either win a given sum of money (a positive number), or lose a given sum of money (a negative number). Both winning and losing have an equal probability, that is, 50 %. You can imagine, for example, that a coin is tossed to determine the outcome. You have to decide whether you accept the gamble presented (←) or reject it (→). For example, if you see on the screen a pair of numbers **1.0 -0.50**, you have to decide whether you accept or reject a gamble that offers a 50% change of winning 1.0 euro or a 50% change of losing 0.50 euro.

At the end of the experiment, you will receive a cash payment. The amount of the payment will depend partly on the decisions that you make. At the end of the experiment (all rounds), we will randomly choose five gambles for actual payment, each gamble with equal likelihood. Among the five gambles, the gambles you accepted during the experiment will be played for real. The outcome will be decided by tossing a coin: heads means you will win, tails means you will lose. At the maximum, you can win 20 euro, or lose 10 euro.

Before starting the experiment, you will be able to practice.

The experiment will last approximately 40 minutes.
